# Analysis of ineffective breathing pattern and impaired spontaneous ventilation of adults with oxygen therapy ^1^

**DOI:** 10.1590/1518-8345.1950.2954

**Published:** 2017-12-04

**Authors:** Deborah Hein Seganfredo, Beatriz Amorim Beltrão, Viviane Martins da Silva, Marcos Venícios de Oliveira Lopes, Stela Maris de Jezus Castro, Miriam de Abreu Almeida

**Affiliations:** 2 PhD, RN, Hospital Nossa Senhora da Conceição, Porto Alegre, RS, BR; 3 PhD, RN, Hospital Universitário Walter Cantídio, Fortaleza, CE, BR; 4 PhD, Adjunct Professor, Nursing Department, Universidade Federal do Ceará, Fortaleza, CE, BR; 5 PhD, Associate Professor, Nursing Department, Universidade Federal do Ceará, Fortaleza, CE, BR; 6 PhD, Adjunct Professor, Departamento de Estatística, Universidade Federal do Rio Grande do Sul, Porto Alegre, RS, BR; 7 PhD, Associate Professor, Nursing School, Universidade Federal do Rio Grande do Sul, Porto Alegre, RS, BR

**Keywords:** Nursing, Nursing Diagnosis, Nursing Process

## Abstract

**Objective:**

to analyze the manifestation of the defining characteristics of the nursing diagnoses of ineffective breathing pattern and impaired spontaneous ventilation, of the NANDA International and the defining characteristics identified in the literature for the concept of “ventilation” in adult patients hospitalized in an intensive care unit with use of oxygen therapy.

**Method:**

clinical diagnostic validation study, conducted with 626 patients in intensive care using oxygen therapy, in three different modalities. Multiple correspondence analysis was used to verify the discriminative capacity of the defining characteristics and latent class analysis to determine the diagnostic accuracy of them, based on the severity level defined by the ventilatory mode used.

**Results:**

in the multiple correspondence analysis, it was demonstrated that the majority of the defining characteristics presented low discriminative capacity and low percentage of explained variance for the two dimensions (diagnoses). Latent class models, separately adjusted for the two diagnoses, presented a worse fit, with sharing of some defining characteristics. Models adjusted by level of severity (ventilation mode) presented better fit and structure of the component defining characteristics.

**Conclusion:**

clinical evidence obtained in the present study seems to demonstrate that the set of defining characteristics of the two nursing diagnoses studied fit better in a single construct.

## Introduction

The NANDA International Taxonomy II (NANDA-I) is the globally accepted classification of Nursing Diagnoses (NDs), composed of 13 domains, 47 classes and 234 diagnoses. The fourth domain, called “Activity/rest”, is defined as the “production, conservation, expenditure or balance of energy resources” and contains the class “Cardiovascular/pulmonary responses”, which is defined as “cardiopulmonary mechanisms that support activity/rest”. This class includes the Ineffective Breathing Pattern (IBP) and Impaired Spontaneous Ventilation (ISV) NDs^1^.

The IBP ND was introduced into the classification in 1980 and was revised three times in the years 1996, 1998 and 2010. It is defined as “inspiration and/or expiration that does not provide adequate ventilation” and has 16 Defining Characteristics (DCs). The ISV ND, in turn, was introduced into the NANDA-I in 1992 and has never been revised. It is defined as “decreased energy reserves resulting in an inability to maintain independent breathing that is adequate to support life” and has 11 DCs. The difficulties in accurately establishing each of these NDs come from both their closely related definitions and their similar or shared DCs. 

In the clinical practice, the ISV ND is often attributed to patients admitted to an Intensive Care Unit (ICU) who are dependent on invasive mechanical ventilation (IMV)^2^. However, this ND is mainly used due to its title, because, when the DCs and related factors are analyzed, both the IBP and the ISV ND could be attributed to the patient on IMV. It is also appropriate to examine the definitions of each of the NDs. The IBP ND presents the words “adequate ventilation” in its definition, while the ISV ND presents the words “adequate breathing”. Therefore, key concepts of IBP ND title are present in the definition of the ISV ND and vice versa. In the analysis of the central concept of IBP and ISV, both probably refer to the concept of “ventilation” and not to the concept of “breathing” present in the IBP title. The ND available for patients with problems in the processes of respiration/exchange of gases in cell membranes is Impaired Gas Exchange (IGE), defined as “excess or deficit in oxygenation and/or carbon dioxide elimination at the alveolar-capillary membrane”^1^ ^,^ ^3^

Therefore it is possible that IBP and ISV respond for the same diagnostic concept, being in fact different levels of severity of the same ND. Thus, the aim of this study was to analyze how the DCs of the IBP and ISV NDs and the DCs found in the literature for the key concept “ventilation” are manifested in adult patients hospitalized in ICU using oxygen therapy.

## Method

This was a cross-sectional, methodological study, for clinical validation between the IBP and ISV NDs. Clinical validation was used to test whether the clinical data (defining characteristics) supported the existence of two distinct NDs, IBP and ISV, or only one ND with the key concept “ventilation”, consisting of different levels of severity. The variable “ventilatory mode” was used to determine the severity levels of the assumed ND with “ventilation” as the key concept. Thus, patients undergoing oxygen therapy using face masks or nasal cannulars, that is, Spontaneous Ventilation (SV), were considered as the lowest severity level, patients submitted to oxygen therapy through Non-Invasive Mechanical Ventilation (NIMV), the level of intermediate severity and the patients submitted to IMV, the level of greater severity.

The present study, performed in the ICU of a federal tertiary hospital that provides general clinical care for adults and is linked to the Brazilian National Health System (SUS), was developed with patients of both sexes, aged 18 years and over, using the following modalities of oxygen therapy: 1) SV, 2) NIMV and 3) IMV. The study included patients hospitalized in the ICU for a maximum of seven days, with the intention of not including patients on prolonged mechanical ventilation, since there is no uniform definition of prolonged intubation in the literature, ranging from 7 and 21 days^4^ ^-^ ^5^. The exclusion criteria established were: 1) to have neurological and/or muscular disease that may cause a change in the clinical presentation of the DCs of the NDs under study and 2) to be using neuromuscular blockers and/or moderate to profound sedation according to the Richmond Sedation and Agitation Scale (RASS)^6^. Subjects were included in the study by consecutive sampling. In studies using the Latent Class Analysis (LCA) technique, the minimum sample size presented is at least 20 observations (individuals) for each item (DC) to be analyzed^7^. Thus, the minimum sample size for 25 DCs equates to 500 patients, with the sample in this study consisting of 626 individuals.

The data collection instrument was developed containing the characterization of the patient, ventilatory data and a table with four columns. The first column contained the DCs found in the analysis of the “ventilation” concept previously performed^8^, as well as the NANDA-I DCs for the IBP and ISV NDs^1^. The second column contained the respective operational definitions of each DC, constructed based on the clinical experience of the researcher, of the specialists and in the literature. The last two columns presented the options “yes” and “no”, regarding the presence of that DC in the physical examination of the patient. For example, the “decrease in inspiratory pressure” DC was operationally defined as inspiratory pressure greater than -90 cmH2O, with the “decrease in expiratory pressure” DC defined as expiratory pressure lower than +100 cmH2O, measured by means of a manometer^9^. However, in the pilot test, it was evidenced that this examination depended on the cooperation and level of consciousness of the patient, which was impaired in the critically ill patients. Thus, 100% consensus was reached among the specialists in assigning these DCs as present in patients requiring positive pressure ventilatory support (IMV and NIMV) and not present in SV patients. Also the “alterations in tidal volume” DC was evaluated when changes were noticed in the depth of the ventilatory movement in SV patients, that is, the increase or decrease in its amplitude. In patients with IMV or NIMV, current volumes greater than or less than 4-8 ml/kg^9^ were considered.

Data collection was carried out, where the study was performed, by the researcher and eight ICU care nurses with the qualification of specialists in intensive care and at least three years of clinical practice in an adult ICU. Data were collected from February 2015 to January 2016. Each patient was evaluated individually by a single nurse, who performed the physical examination and filled in the data collection instrument based on this clinical evaluation and data from the medical chart of the patient.

A pilot test was performed with 30 patients, in order to test the applicability of the instrument and refine it. Thus, the evaluating nurses recorded the presence or absence of each of the 38 DCs listed on the pilot test instrument. Next, the instrument was refined in a meeting with the specialist nurses, which resulted in 25 DCs, in its final version. The univariate descriptive analysis was presented through measures of absolute frequency, percentage, central tendency and dispersion. The statistical analysis was performed using the Statistical Package for the Social Sciences (SPSS), version 20, for Macintosh, and the R software, version 3.4.0. The Multiple Correspondence Analysis (MCA) was used to identify the discriminative capacity of each DC for the IBP and ISV NDs, with the intention of performing the differential diagnosis^7^. The LCA with random effects was used to calculate the sensitivity and specificity of the DCs of the ND in question^7^. For defining the DCs to be included in these models, sensitivity and/or specificity values ​​were defined with higher confidence intervals, the lower band of which was above 0.5 (50%). The significance level of 5% was adopted for all analyses.

The research project was approved, under Authorization No. 14295, by the Research Ethics Committee of the Conceição Hospital Group, and registered in the *Plataforma Brasil* with CAAE No. 40366114.9.0000.5530, fulfilling the national and international ethical requirements for research with human subjects, following Resolution 466/2012.

## Results

Table 1 presents the results regarding the characterization of the sample of patients submitted to the clinical bedside evaluation in the ICU.

**Table 1:** Characterization of the sample. Porto Alegre, RS, Brazil, 2016

**Variables**		**Mean**	**Standard Deviation**	
**Age**		**59,02**	**17,64**	
		N*=626	**Percentage**	**95% CI**
**Sex**				
	**Male**	**335**	**53.5**	**49.6-57.5**
	**Female**	**291**	**46.5**	**42.5-50.5**
**Ventilatory mode**				
	**Spontaneous ventilation**	**323**	**51.6**	**47.6-55.6**
	**Non-invasive mechanical ventilation**	**128**	**20.5**	**17.4-23.9**
	**Invasive mechanical ventilation**	**175**	**27.9**	**24.5-31.7**
**Sedation**				
	**Yes**	**115**	**18.4**	**15.4-21.7**
	**No**	**511**	**81.6**	**78.3-84.5**
**Reason for the need for oxygen therapy**				
	**Acute repertory insufficiency**	**626**	**100.0**	**--**
	**Hypoxemia**	**539**	**86.1**	**83.1-88.7**
	**Hypercapnia**	**87**	**13.9**	**11.3-16.9**

*Number of patients

In the analysis of the main clinical characteristics, there was a similarity of proportions by sex and a predominance of patients on SV, followed by patients on IMV. The three groups presented statistically different proportions (see 95% CI in Table 1).

Table 2 presents the absolute frequencies and percentages of DCs observed in the clinical bedside examination.

**Table 2:** Distribution of the defining characteristics for the ineffective breathing pattern and impaired spontaneous ventilation diagnoses, as well as for the review of the “ventilation” concept for the total sample of patients and subsamples related to the type of ventilatory support. Porto Alegre, RS, Brazil, 2016

**Defining characteristics**	Total (N*=626) **f** ^**†**^ **%** ^**‡**^		**SV** ^**§**^ **(n=323)** **f %**		**NIMV** ^**||**^ **(n=128)** **f %**		**IMV** ^**¶**^ **(n=175)** **f %**	
**Alterations in respiratory rate**	**400**	**63.6**	**189**	**58.4**	**98**	**76.4**	**113**	**64.4**
**Decrease in inspiratory pressure**	**309**	**49.1**	**6**	**1.9**	**128**	**100**	**175**	**100**
**Decrease in expiratory pressure**	**309**	**49.1**	**6**	**1.9**	**128**	**100**	**175**	**100**
**Increase in heart rate**	**293**	**46.6**	**126**	**38.9**	**82**	**63.9**	**85**	**48.4**
**Use of accessory muscles to breathe**	**288**	**45.8**	**125**	**38.6**	**90**	**70.2**	**73**	**41.6**
**Altered arterial blood gases**	**288**	**45.8**	**93**	**28.7**	**85**	**66.3**	**110**	**62.7**
**Increased metabolic rate**	**282**	**44.8**	**117**	**36.2**	**81**	**63.1**	**84**	**47.8**
**Dyspnea**	**251**	**40.0**	**152**	**47.0**	**49**	**38.2**	**50**	**28.5**
**Alterations in tidal volume**	**224**	**35.6**	**36**	**11.1**	**88**	**68.6**	**100**	**57.0**
**Fatigue**	**220**	**35.0**	**97**	**30.0**	**65**	**50.7**	**58**	**33.0**
**Orthopnea**	**210**	**33.4**	**142**	**43.9**	**35**	**27.3**	**33**	**18.8**
**Abdominal paradoxical respiratory pattern**	**205**	**32.6**	**123**	**38.0**	**26**	**20.2**	**56**	**31.9**
**Increased restlessness**	**201**	**32.0**	**78**	**24.1**	**54**	**42.1**	**69**	**39.3**
**Altered ventilation/perfusion ratio**	**179**	**28.5**	**51**	**15.7**	**52**	**40.5**	**76**	**43.3**
Decrease in SaO2**	**161**	**25.6**	**13**	**4.0**	**64**	**49.9**	**84**	**47.8**
**Decrease in cooperation**	**133**	**21.1**	**41**	**12.6**	**39**	**30.4**	**53**	**30.2**
**Hypoxia**	**114**	**18.1**	**30**	**9.2**	**31**	**24.1**	**53**	**30.2**
**Apprehensiveness**	**86**	**13.7**	**35**	**10.8**	**28**	**21.8**	**23**	**13.1**
**Cyanosis of the skin, lips or extremities**	**65**	**10.3**	**15**	**4.6**	**11**	**8.5**	**39**	**22.2**
**Prolonged expiration phase**	**58**	**9.2**	**34**	**10.5**	**10**	**7.8**	**14**	**7.9**
**Use of three-point position**	**40**	**6.4**	**8**	**2.4**	**23**	**17.9**	**9**	**5.1**
**Increase in anterior-posterior chest diameter**	**26**	**4.1**	**11**	**3.4**	**7**	**5.4**	**8**	**4.5**
**Nasal flaring**	**11**	**1.7**	**3**	**0.9**	**3**	**2.3**	**3**	**1.7**
**Drumming of fingers**	**9**	**1.4**	**5**	**1.5**	**5**	**3.9**	**1**	**0.5**
**Altered chest excursion**	**1**	**0.2**	**0**	**0**	**0**	**0**	**1**	**0.5**

*Number of patients; †Frequency; ‡Percentage; §Spontaneous ventilation; ||Non-invasive mechanical ventilation; ¶Invasive mechanical ventilation; **Arterial oxygen saturation

Next, the results obtained from the MCA and LCA techniques are presented. Table 3 presents the results obtained from the MCA technique.

**Table 3:** Discrimination measures for the two dimensions obtained by multiple correspondence analysis, for the total sample of patients and subsamples related to the type of ventilatory support. Porto Alegre, RS, Brazil, 2016

**Defining characteristics**	**Total** (N*=626)		**IMV** ^**†**^ **(n=175)**		**NIMV** ^**‡**^ **(n=128)**		**SV§** **(n=323)**	
	**1**	**2**	**1**	**2**	**1**	**2**	**1**	**2**
**Nasal flaring**	**0.007**	**0.003**	**0.002**	**0.001**	**0.005**	**0.018**	**0.020**	**0.147**
**Use of three-point position**	**0.054**	**0.007**	**0.071**	**0.158**	**0.001**	**0.018**	**0.016**	**0.055**
**Increase in anterior-posterior chest diameter**	**0.014**	**0.006**	**0.124**	**0.069**	**0.000**	**0.027**	**0.000**	**0.000**
**Orthopnea**	**0.208**	**0.011**	**0.150**	**0.009**	**0.236**	**0.045**	**0.282**	**0.133**
**Altered chest excursion**	**0.001**	**0.007**	**0.001**	**0.003**	**--**	**--**	**--**	**--**
**Prolonged expiration phase**	**0.008**	**0.017**	**0.034**	**0.020**	**0.008**	**0.039**	**0.046**	**0.012**
**Alterations in repertory rate**	**0.266**	**0.324**	**0.510**	**0.022**	**0.488**	**0.198**	**0.502**	**0.031**
**Decrease in inspiratory pressure**	**0.483**	**0.101**	**--**	**--**	**--**	**--**	**0.027**	**0.595**
**Decrease in expiratory pressure**	**0.487**	**0.099**	**--**	**--**	**0.061**	**0.000**	**0.027**	**0.595**
**Apprehensiveness**	**0.032**	**0.003**	**0.007**	**0.000**	**0.001**	**0.000**	**0.067**	**0.129**
**Decrease in cooperation**	**0.179**	**0.230**	**0.038**	**0.573**	**0.082**	**0.550**	**0.120**	**0.000**
**Increase in heart rate**	**0.320**	**0.399**	**0.541**	**0.083**	**0.637**	**0.207**	**0.529**	**0.031**
**Increased restlessness**	**0.197**	**0.155**	**0.119**	**0.401**	**0.059**	**0.611**	**0.175**	**0.000**
**Altered arterial blood gases**	**0.375**	**0.025**	**0.246**	**0.084**	**0.256**	**0.090**	**0.158**	**0.075**
**Decrease in SaO2** ^**||**^	**0.353**	**0.095**	**0.095**	**0.152**	**0.083**	**0.078**	**0.047**	**0.224**
**Increased metabolic rate**	**0.333**	**0.387**	**0.536**	**0.080**	**0.669**	**0.200**	**0.505**	**0.033**
**Use of accessory muscles to breathe**	**0.155**	**0.240**	**0.434**	**0.115**	**0.097**	**0.259**	**0.141**	**0.002**
**Fatigue**	**0.088**	**0.117**	**0.311**	**0.067**	**0.004**	**0.042**	**0.064**	**0.026**
**Cyanosis of the skin, lips or extremities**	**0.137**	**0.014**	**0.273**	**0.012**	**0.027**	**0.008**	**0.053**	**0.023**
**Drumming of fingers**	**0.006**	**0.005**	**0.036**	**0.004**	**0.012**	**0.011**	**0.003**	**0.004**
**Abdominal paradoxical respiratory pattern**	**0.072**	**0.026**	**0.036**	**0.020**	**0.050**	**0.002**	**0.117**	**0.055**
**Alterations in tidal volume**	**0.341**	**0.050**	**0.070**	**0.047**	**0.080**	**0.001**	**0.043**	**0.107**
**Altered ventilation/perfusion ratio**	**0.350**	**0.025**	**0.333**	**0.141**	**0.303**	**0.046**	**0.135**	**0.027**
**Hypoxia**	**0.220**	**0.161**	**0.036**	**0.572**	**0.303**	**0.362**	**0.122**	**0.016**
**Dyspnea**	**0.130**	**0.015**	**0.074**	**0.007**	**0.135**	**0.040**	**0.271**	**0.135**
**Total active**	**4.81**	**2.52**	**4.07**	**2.64**	**3.59**	**2.85**	**3.47**	**2.45**
**Percentage of variance**	**19.2**	**10.0**	**17.7**	**11.4**	**15.6**	**12.3**	**14.4**	**10.2**
**Cronbach’s alpha**	**0.82**	**0.62**	**0.78**	**0.64**	**0.75**	**0.67**	**0.74**	**0.61**

*Number of patients; †Invasive mechanical ventilation; ‡Non-invasive mechanical ventilation; §Spontaneous ventilation; ||Arterial oxygen saturation

When evaluating the capacity of the DCs for the discrimination of two assumed dimensions, it was identified that, among the 25 DCs evaluated, eight (32%) presented low discriminative values ​​for the total sample, for IMV and for SV. Patients on NIMV presented, in total, 12 DCs (48%) with low discriminative capacity. In general, 14 DCs (56%) presented low measures of discrimination for at least one of the samples analyzed. Among the 12 DCs with high measures of discrimination for at least one of the two dimensions, only the “orthopnea”, “altered blood gases”, and “altered ventilation/perfusion ratio” DCs presented consistently higher measures for the same dimension. Another four DCs (“hypoxia”, “use of accessory muscles to breathe”, “increased restlessness” and “decrease in cooperation”) showed measures of discrimination with alternation of high values ​​between the two dimensions, in the analysis of the three levels of ventilatory support. In addition, three DCs (“increased metabolic rate”, “increase in heart rate” and “alterations in respiratory rate”) presented high values ​​for one dimension, in the patient subsamples, and high values ​​for the other, when considering the total sample. Finally, the percentage of explained variance was less than 30%, with the internal consistency values ​​being lower than 0.7 for the second dimension, in all samples. Table 4 presents the measures of diagnostic accuracy obtained by LCA, from the DCs of the IBP and ISV NDs.

**Table 4:** Measures of diagnostic accuracy obtained by latent class analysis, with random effects, adjusted from the specific defining characteristics of the ineffective breathing pattern and impaired spontaneous ventilation diagnoses. Porto Alegre, RS, Brazil, 2016

**Defining characteristics**	Se*	**95% CI** ^**†**^		**Sp** ^**‡**^	**95% CI** ^**†**^	
**1. IBP** ^**§**^						
**Drumming of fingers**	**0.022**	**0.010**	**0.059**	**0.987**	**0.808**	**0.998**
**Fatigue**	**0.407**	**0.358**	**0.459**	**0.703**	**0.648**	**0.752**
**Cyanosis of the skin, lips or extremities**	**0.161**	**0.127**	**0.208**	**0.952**	**0.921**	**0.970**
**Use of three-point position**	**0.106**	**0.076**	**0.148**	**0.977**	**0.950**	**0.989**
**Increase in anterior-posterior chest diameter**	**0.048**	**0.030**	**0.081**	**0.965**	**0.927**	**0.981**
**Altered chest excursion**	**0.003**	**0.000**	**0.995**	**1.000**	**1.000**	**1.000**
**Prolonged expiration phase**	**0.081**	**0.056**	**0.118**	**0.895**	**0.853**	**0.924**
**Abnormal breathing pattern**	**0.692**	**0.638**	**0.736**	**0.413**	**0.361**	**0.462**
**Alterations in tidal volume**	**0.617**	**0.564**	**0.671**	**0.895**	**0.852**	**0.926**
**Altered ventilation/perfusion ratio**	**0.416**	**0.365**	**0.479**	**0.842**	**0.791**	**0.876**
**Hypoxia**	**0.274**	**0.225**	**0.334**	**0.908**	**0.868**	**0.932**
**Decrease in inspiratory pressure**	**1.000**	**1.000**	**1.000**	**1.000**	**1.000**	**1.000**
**Decrease in expiratory pressure**	**0.996**	**0.000**	**0.999**	**1.000**	**1.000**	**1.000**
**Prevalence**	**49.4%**		**G** ^**2||**^ **: 653.5**	**DF** ^**¶**^ **: 599**		p**=0.061
**2. ISV** ^**††**^						
**Cyanosis of the skin, lips or extremities**	**0.216**	**0.158**	**0.283**	**0.963**	**0.917**	**0.982**
**Apprehensiveness**	**0.182**	**0.136**	**0.244**	**0.889**	**0.846**	**0.920**
**Altered arterial blood gases**	**0.858**	**0.737**	**0.917**	**0.775**	**0.629**	**0.865**
**Hypoxia**	**0.365**	**0.291**	**0.444**	**0.926**	**0.879**	**0.954**
**Decrease in SaO2** ^**‡‡**^	**0.681**	**0.520**	**0.797**	**0.993**	**0.001**	**0.999**
**Prevalence**	**37.2%**		**G** ^**2**^ **: 29.6**	**DF: 20**		**P=0.077**

*Sensitivity; †95% Confidence Interval; ‡Specificity; §Ineffective breathing pattern; ||G2 Statistic of the likelihood ratio; ¶Degrees of freedom; **Level of significance; ††Impaired spontaneous ventilation; ‡‡Arterial oxygen saturation

The models adjusted separately for IBP and ISV, and including only the DCs described for each ND, together with the DCs identified in the review, did not present a good fit, bordering the level of significance adopted. More DCs were included in the IBP model (13) compared to the DCs included in the ISV model (5). Table 5 presents the measures of diagnostic accuracy obtained by LCA, from all the DCs of the IBP and ISV NDs, in the subsamples of patients.

**Table 5:** Measures of diagnostic accuracy obtained by latent class analysis, with random effects, adjusted from the all the defining characteristics of the ineffective breathing pattern and impaired spontaneous ventilation diagnoses, in the subsamples of patients. Porto Alegre, RS, Brazil, 2016

**Defining characteristics**		Se*	**95% CI** ^**†**^		**Sp** ^**‡**^	**95% CI** ^**†**^	
**1. Patients on spontaneous ventilation (n** ^**§**^ **=323)**							
	**Fatigue**	**0.500**	**0.001**	**0.996**	**0.703**	**0.649**	**0.754**
	**Increased restlessness**	**0.332**	**0.000**	**0.999**	**0.760**	**0.711**	**0.806**
	**Prolonged expiration phase**	**0.165**	**0.000**	**0.999**	**0.895**	**0.859**	**0.924**
	**Alterations in tidal volume**	**0.500**	**0.002**	**0.997**	**0.895**	**0.855**	**0.926**
	**Decrease in inspiratory pressure**	**1.000**	**0.999**	**1.000**	**1.000**	**1.000**	**1.000**
	**Decrease in expiratory pressure**	**1.000**	**0.999**	**1.000**	**1.000**	**1.000**	**1.000**
	**Decrease in SaO2** ^**||**^	**0.332**	**0.000**	**0.999**	**0.965**	**0.933**	**0.979**
	**Prevalence**	**1.9%**		**G** ^**2¶**^ **: 51.6**	DF**: 112		**p** ^**††**^ **=0.999**
**2. Patients on noninvasive mechanical ventilation (n=128)**							
	**Use of three-point position**	**0.211**	**0.134**	**0.324**	**0.855**	**0.696**	**0.932**
	**Decrease in cooperation**	**0.377**	**0.270**	**0.520**	**0.776**	**0.612**	**0.877**
	**Abnormal breathing pattern**	**0.843**	**0.689**	**0.925**	**0.321**	**0.210**	**0.488**
	**Alterations in tidal volume**	**0.848**	**0.692**	**0.930**	**0.492**	**0.361**	**0.644**
	**Altered ventilation/perfusion ratio**	**0.770**	**0.002**	**0.997**	**1.000**	**1.000**	**1.000**
	**Altered arterial blood gases**	**1.000**	**1.000**	**1.000**	**0.711**	**0.002**	**0.995**
	**Decrease in expiratory pressure**	**1.000**	**1.000**	**1.000**	**0.016**	**0.000**	**0.999**
	**Prevalence**	**52.8%**		**G** ^**2**^ **: 44.3**	**DF: 112**		**p=1.000**
**3. Patients on invasive mechanical ventilation (n=175)**							
	**Cyanosis of the skin, lips or extremities**	**0.227**	**0.129**	**0.411**	**0.779**	**0.689**	**0.843**
	**Use of three-point position**	**0.000**	**0.000**	**0.000**	**0.926**	**0.858**	**0.961**
	**Apprehensiveness**	**0.189**	**0.108**	**0.350**	**0.894**	**0.801**	**0.941**
	**Decrease in cooperation**	**0.999**	**0.724**	**1.000**	**1.000**	**0.999**	**1.000**
	**Increased restlessness**	**0.866**	**0.370**	**0.976**	**0.812**	**0.680**	**0.876**
	**Abnormal breathing pattern**	**0.715**	**0.586**	**0.819**	**0.384**	**0.302**	**0.475**
	**Altered arterial blood gases**	**0.659**	**0.512**	**0.782**	**0.384**	**0.307**	**0.478**
	**Hypoxia**	**0.753**	**0.559**	**0.867**	**0.894**	**0.787**	**0.938**
	**Prevalence**	**30.3%**		**G** ^**2**^ **: 150.8**	**DF: 158**		**p=0.644**

*Sensitivity; †95% Confidence Interval; ‡Specificity; §Number of patients; ||Arterial oxygen saturation; G2 Statistic of the likelihood ratio; **Degrees of freedom; ††Level of significance

When the models were adjusted for each of the three subsamples of patients, on different types of ventilatory supports, 15 DCs, in total, presented good diagnostic accuracy values ​​for at least one of them. All the models included DCs specific for IBP, for ISV and common to the two NDs or identified in the review of the “ventilation” concept. In addition to the models presented, models were adjusted with all the DCs together, without distinction of ND, considering each ND separately and without severity levels, with none of them presenting good fits. Thus, in this study, the best models were identified when treating the set of DCs as members of a single ND.

## Discussion

Considering the aims of this study, namely, to analyze how the DCs of the IBP and ISV NDs are manifested, as well as those contained in the literature for the key concept “ventilation” in adult patients hospitalized in an ICU, using oxygen therapy, it was found that “decreased inspiratory pressure” and “decreased expiratory pressure” presented the highest frequency, in both (100%) the IMV and NIMV subsamples of patients. This fact is attributed to the need for the patients of both of the severity subsamples to receive oxygen therapy with positive pressure in the airways. However, in the NIMV therapy, the patients need to use their muscles to ventilate, this being a facilitator of the respiratory work and not a therapy to substitute the ventilatory musculature^10^ ^-^ ^11^. Thus, this may explain why the “decrease in inspiratory pressure”, “decrease in expiratory pressure”, “increase in heart rate”, “alterations in respiratory rate”, “increased metabolic rate”, “use of accessory muscles to breathe”, “altered arterial blood gases” and “alterations in tidal volume” DCs presented higher frequencies for the intermediate severity sub-sample, compared to the greater severity sub-sample. Therefore, in the patients of greater severity, on IMV oxygen therapy, the mechanical ventilator can completely replace the work of the ventilatory musculature, causing the patient to present a lower number of DCs. In the patients with more severe morbidity spectra, IMV can increase survival and provide the necessary support for oxygenation, while the body recovers from a serious injury^12^.

Comparing the DCs that obtained better measures of diagnostic accuracy in the different severity subscales, it is evident that there are DCs common to more than one subsample. The DCs “altered arterial blood gases”, “alterations in respiratory rate”, “use of three-point position” and “decrease in cooperation” obtained high sensitivity values ​​for the NIMV and IMV patient subsamples and may be associated with later states of injury to the respiratory system, in which the mechanisms of compensation of the organism are not sufficient to compensate for the imbalance of blood gases and, consequently, of the pH^9^ ^,^ ^13^. Thus, in these subsamples, altered states of consciousness can be evidenced due to the decrease in PO2 and consequent hypoxia, leading patients to present “decrease in cooperation”. However, individuals who do not require positive pressure ventilation therapies usually present better clinical conditions and, respectively, lower levels of severity, since their ventilatory muscles are able to overcome the pressure demand necessary for inspiratory and expiratory ventilation movements. In these patients, the “alterations in respiratory rate”, “dyspnea” and “orthopnea” DCs may be the first clinical indications that there is progressing ventilatory dysfunction^3^. In the MCA, it was shown that the division of the DCs into two dimensions, that is, into two nursing diagnoses, is relatively inconsistent, with a low percentage of explained variance. Thus, the data indicates that to consider that these DCs represent the IBP and ISV NDs is inadequate, both for the total sample and for the subsamples related to the types of ventilatory support.

To confirm these findings, latency class models with random effects were adjusted for all the DCs representing a single ND and for the two sets of DCs, ISV and IBP, separately. After the analyses in question were repeated for the whole sample and for the ventilatory support subsamples the results showed that, when considering the existence of a single ND, the latent class models presented a good fit and included DCs from ISV, IBP and the analysis of the “ventilation” concept, indicating greater consistency of a single ND. On the other hand, the models for the NDs, adjusted separately, showed worse fit. Also, some of the DCs found in the final models, in particular for ISV, did not compose the ND described in the NANDA-I and were included in the models because they were identified in the review of the “ventilation” concept. Therefore, the data obtained from the LCA corroborate what was found in the MCA, that is, there is evidence that the set of DCs studied contemplates a single ND, with three clinical spectra associated with the type of ventilatory support. To ratify this finding, in the latent class model adjusted separately for the two NDs, and including the DCs described for each ND along with the DCs identified in the concept analysis, the DCs that appeared in the ISV were “cyanosis of the skin, lips or extremities”, “apprehensiveness”, “altered arterial blood gases”, “hypoxia” and “decrease in SaO2”. However, it is highlighted in the literature that these DCs may indicate the presence of the IGE ND^3^. These DCs demonstrate impairment of pulmonary gas exchange function, not pulmonary ventilation processes. With this, they are observed later, when the mechanisms of compensation of the respiratory system are exhausted. Hypoxia may be preceded by signs of physiological compensation for respiratory stress, including “use of accessory muscles to breathe” and “alterations in respiratory rate”. After this stage of compensation, the DCs “apprehensiveness”, “altered arterial blood gases”, “hypoxia” and “decrease in SaO2” can be evidenced, characterizing the TGP ND^3^ ^,^ ^13^.

The DCs that presented higher sensitivity values ​​for the SV subsample, in the latent class model with better fit were “decrease in inspiratory pressure”, “decrease in expiratory pressure”, “fatigue”, and “decrease in SaO2”. In one study, among the main DCs evidenced to predict the IBP ND in children with acute respiratory infection, there were “use of accessory muscles to breathe” and “dyspnea”, in which high sensitivity (88.84% and 86.78%) and specificity (99.53% and 86.18%) values ​​were observed^14^. In the present study, these DCs were not included in the characteristics with higher sensitivities. This fact can be justified because the sample of the study cited consisted of children, not adults. However, it should be highlighted that all the patients included in the present study already had oxygen therapy instituted, a fact that may have mitigated the presentation of the DCs studied or even suppressed them, considering that the oxygen supply above the normal atmospheric concentration (>21%) for any of the ventilatory modalities, i.e. SV, NIMV or IMV, may have compensated for the cause of ventilatory impairment.

In the intermediate severity subsample (NIMV), the DCs with thtae highest sensitivity values ​​were “altered arterial blood gases”, “decrease in expiratory pressure”, “alterations in respiratory rate”, “alterations in tidal volume”, and “altered ventilation/perfusion ratio”. These DCs present the signs indicated in the literature as clinical indicators in patients who are candidates for the use of NIMV^4^. Thus, in the absence of contraindication, it is recommended that, in patients with the inability to maintain spontaneous ventilation, characterized by volume-minute>4Lpm, PaCO2<50 mmHg and pH>7.25, the use of NIMV should be initiated with two pressure levels, with sufficient inspiratory pressure to maintain adequate ventilation to prevent progression to muscle fatigue and/or respiratory arrest^4^.

In the most severe subsample (IMV), the DCs with the highest sensitivity values ​​were “decrease in cooperation”, “increase in restlessness”, “alterations in respiratory rate”, “altered arterial blood gases” and “hypoxia”. In another study, “abnormal arterial gases” were present in 82.8% of adult patients on IMV with the IBP ND, “abnormal respiratory rate” in 77.6% and “hypoxemia” in 62.1%, demonstrating agreement with the findings of the present study, insofar as they are important DCs for patients with ventilatory dysfunction supported with IMV^15^. Measures of diagnostic accuracy were not calculated, and ISV was not studied because the author understood that this ND was not related to mechanically ventilated patients.

The “tachycardia” CD (44.8%) presented a frequency similar to that reported in the present study (“increase in heart rate”), both for the total sample of patients (46.6%) and for the subsample of patients on IMV (48.4%). The DCs “decrease in inspiratory pressure” and “decrease in expiratory pressure” were the most frequent in the present study for the subsample of patients on IMV (100%), however, in the study cited only “decrease in inspiratory pressure” (24.1%%) was presented by the patients, occupying the fifth position in relation to the IBP ND. On the other hand, the “decreased expiratory pressure” CD was not among the most frequent^15^.

As a limitation in this study, it should be highlighted that all patients included in the sample already had oxygen therapy instituted, in addition to the therapeutic support available in the tertiary level ICU, in which the study was performed. This fact may have mitigated the presentation of the DCs studied or even suppressed them, due to the possible compensation of the cause of the ventilatory impairment.

## Conclusion

Clinical evidence, obtained in the present study, seems to demonstrate that the set of DCs of the two NDs studied fit best in a single construct. One of the possibilities would be the incorporation of ISV DCs into the IBP ND, which constituted part of the latent class model with better fit, i.e. “increase in restlessness”, “changes in tidal volume”, “decreased SaO2”, “decreased cooperation”, “Altered arterial blood gases”, “apprehensiveness” and “hypoxia”. Other DCs, with worse performance in the measures of diagnostic accuracy for each of the severity subsamples, may represent minor DCs for the determination of the IBP ND, with their maintenance in the NANDA-I requiring new studies in order to verify their suitability. Finally, the need to develop studies similar to the present study in distinct populations is reinforced, in order to establish a comparison between the measures of diagnostic accuracy of the DCs. With data from different studies it will be possible to generate higher levels of evidence for the NDs of the NANDA-I.
